# Editorial: Application of Plant Secondary Metabolites to Pain Neuromodulation

**DOI:** 10.3389/fphar.2020.623399

**Published:** 2021-01-14

**Authors:** Rajeev K. Singla, Adriana Gibara Guimarães, Gokhan Zengin

**Affiliations:** ^1^ Institutes for Systems Genetics, Frontiers Science Center for Disease-Related Molecular Network, West China Hospital, Sichuan University, Chengdu, China; ^2^ Federal University of Sergipe, São Cristóvão, Brazil; ^3^ Department of Biology, Faculty of Science, Selcuk University Campus, Konya, Turkey

**Keywords:** pain, nociception, natural products, secondary metabolites, phytochemicals, medicinal plants

**Editorial on the Research Topic**

**Application of Plant Secondary Metabolites to Pain Neuromodulation**

Pain is a highly unpleasant and intolerable condition, which is associated with multiple diseases and disorders, including but not limited to cancer, diabetes, infectious diseases, neurological and dysfunctional disorders, etc (Li et al., 2019; Yang, 2019; Singla et al., 2020). Further, tissue damage or chronic disease of the somatosensory nervous system cause neuropathic pain, which impacts and disturbs life to a higher extent (Pu et al., 2019; Finnerup et al., 2020). Natural resources especially plants have served and contributed several potential drugs for the alleviation and treatment of pain, either directly or in the derived form (Singla et al., 2018; Santos et al., 2019). For instance, the standard drugs like morphine, cannabidiol, acetylsalicylic acid are some of the key examples gifted by the nature. Even regular and healthy dietary food contains many phenolic compounds that elicit the potential to be anti-inflammatory (Laganà et al., 2019). Thus, in the present research topic, we further emphasized and collected the articles which fill the knowledge gap in this domain.

Brugnatelli et al. (2020) in their article “Irritable Bowel Syndrome: Manipulating the Endocannabinoid System as First-Line Treatment” described the role of the endocannabinoid system (ECS) in irritable bowel syndrome as ECS controls the gut homeostasis and explained how it is an efficient target to do the first-line treatment (Russo, 2016; Zhang et al., 2020). They briefed about the endocannabinoids like anandamide, 2-arachidonoyl glycerol, etc where the former was regulating the appetite and energy balancing while the latter was more involved in the general hunger signal (Di Marzo and Matias, 2005; Acharya et al., 2017). Further, they have also elaborated why menthol, an important phytoconstituent of peppermint oil is effective in IBS treatment.

Uddin et al. (2020) have reviewed the potential of flavonoids for the treatment of neuropathic pain and covered it in their article “Exploring the Promise of Flavonoids to Combat Neuropathic Pain: From Molecular Mechanisms to Therapeutic Implications”. They have comprehensively reviewed and documented how various flavonoids carries the potential to decrease and alleviate various neuropathic pain like that of diabetic neuropathy, chemotherapy-induced peripheral neuropathy, spared nerve injury, thermal hyperalgesia, sciatic nerve ligation-induced neuropathic pain, and sciatic nerve chronic constriction injury. They cited that flavonoids are multimodal and act by different mechanisms viz. inhibiting the reduction of antioxidant defense, decreasing oxidative stress, inhibiting PARP over-activation, inhibiting cellular injury and mitochondrial dysfunction processes, and inhibiting glial cells activation and neuroinflammation.

Jin et al. (2020) in their research article “Lipoxin A4 Inhibits NLRP3 Inflammasome Activation in Rats With Non-compressive Disc Herniation Through the JNK1/Beclin-1/PI3KC3 Pathway” have studied the lipoxin modulated molecular mechanisms associated with inflammation in female Sprague-Dawley rats having non-compressive disc herniation. The test analog of lipoxin, LXA4 was compared with standard LY294002 (phosphoinositide-3 kinase (PI3K) inhibitor) alone or in combination with the test drug. Results indicated that LXA4 was a potential agent leading to an increase in the pain threshold, decrease in the proinflammatory cytokines like TNF-α, IL-1β, and IL-18, while increasing the anti-inflammatory mediators like IL-4, IL-10, and TGF-β as well as autophagy-related proteins like MAP1LC3B, Beclin-1, and PI3KC3.

Boccella et al. in their research article “Treatment With 2-Pentadecyl-2-Oxazoline Restores Mild Traumatic Brain Injury-Induced Sensorial and Neuropsychiatric Dysfunctions” evaluated the effect of 2-pentadecyl-2-oxazoline (PEA-OXA) which is a natural product, on the mild traumatic brain injury (mTBI) induced in the male C57BL/6J mice. PEA-OXA was found to be the adrenergic α-2 antagonist, with the potential to restore and reverse all the effects of mTBI i.e. behavioral changes like depression, the cortical GABA levels, and neuronal activity.

Chia et al. in their research article “Zerumbone Modulates α2A-Adrenergic, TRPV1, and NMDA NR2B Receptors Plasticity in CCI-Induced Neuropathic Pain *In Vivo* and LPS-Induced SH-SY5Y Neuroblastoma *In Vitro* Models”, have studied the neuropathic pain-alleviating effects of zerumbone which is a bioactive compound obtained from the rhizome of *Zingiber zerumbet* (family Zingiberaceae). They reported that zerumbone is a multimodal molecule that elicits its anti-allodynic and antihyperalgesic effects by acting on various receptors viz. TRPV1, NMDA, α-1 adrenoreceptor, α-2 adrenoreceptor, β-1 adrenoreceptor, β-2 adrenoreceptor, and NR2B. They previously documented and validated the zerumbone involvement in the serotonergic system also.

Argueta et al. in their article “A Balanced Approach for Cannabidiol Use in Chronic Pain” briefly documented the use of cannabidiol in the treatment of chronic pain. Cannabidiol, contrary to the tetrahydrocannabinol which was another major metabolite of Cannabis sativa, is a non-psychostimulant molecule and evidently recorded its potential use in intractable chronic pain. As the cannabidiol also possessed teratogenic effects proven through the preclinical studies as well as devoid of any long-term studies, authors recommended that the public should use a balanced approach while dealing with cannabidiol and should avoid any sort of drug abuse.

Assis et al. in their article “Antinociceptive Activity of Chemical Components of Essential Oils That Involves Docking Studies: A Review”, have systemically reviewed the computational studies which had been conducted on essential oil's chemicals for their detailed analysis of antinociceptive potential. The data was extracted from Science Direct and PubMed. They have categorized the various antinociceptive chemicals, software used for evaluation, as well as the molecular targets and their interacting amino acids for eliciting the antinociceptive potential.

Uddin et al. have contributed another review article “Emerging Promise of Cannabinoids for the Management of Pain and Associated Neuropathological Alterations in Alzheimer's Disease” where they have comprehensively reviewed the potential and applications of various cannabinoids for pain management especially in case of Alzheimer's disease (AD). They cited in their article how pain via cascade pathways initiated at the locus coeruleus-noradrenaline system can lead to neuronal death and Alzheimer's disease. Further, they have well elaborated on how cannabinoids are functional in tackling various pathological conditions of AD.

Muñoz-Montesino et al. in their research article “Inhibition of the Glycine Receptor alpha 3 Function by Colchicine” have studied the potential inhibitory effect of colchicine and its mechanism involved while inhibiting the ion channel, α3 subunit based glycine receptor (α3GlyRs) which is primarily involved in the chronic inflammatory pain. They found that the orthosteric site of the α3GlyR's closed state is the main binding site for this colchicine which was found to be the competitive antagonist for the target receptor.

Alberto et al. in the article “Molecular Modeling Applied to the Discovery of New Lead Compounds for P2 Receptors Based on Natural Sources” have comprehensively covered various natural products against P2Y and P2X classes of P2 purinergic receptors as well as elaboratively explained the potential role of various *in silico* tools in achieving the goals.

Singla et al. in their research article “Regulation of Pain Genes- Capsaicin vs Resiniferatoxin: Reassessment of Transcriptomic Data”, have bioinformatically reassessed the transcriptomic data covering the gene regulatory information of two natural products, capsaicin and resiniferatoxin which was earlier published by Isensee et al., (2014). They have found that resiniferatoxin was regulating more non-pain associated genes as compared to capsaicin when the filtering of the genes was done by two pain gene databases.

This research topic, thus covered one brief research report, one mini-review, one opinion, four original research, three reviews, and one systematic review article. In conclusion, it is indeed very clear that plant secondary metabolites are highly efficient in neuromodulating pain via multimodal pathways. Exhaustive exploration in the next step can lead to the development of more potent drugs with the least or minimal side effects.

## Authors Contributions

RS, AG, and GZ have collectively conceived and wrote the text. All authors contributed to the article and approved the submitted version.

## Conflict of Interest

The authors declare that the research was conducted in the absence of any commercial or financial relationships that could be construed as a potential conflict of interest.

## References

[B1] Acharya N. Penukonda S. Shcheglova T. Hagymasi A. T. Basu S. Srivastava P. K. (2017). Endocannabinoid system acts as a regulator of immune homeostasis in the gut. Proc. Natl. Acad. Sci. U.S.A. 114, 5005–5010. 10.1073/pnas.1612177114 28439004 PMC5441729

[B16] Brugnatelli V. Turco F. Freo U. Zanette G. (2020). Irritable bowel syndrome: manipulating the endocannabinoid system as first-line treatment. Front. Neurosci. 14, 371 10.3389/fnins.2020.00371 32372912 PMC7186328

[B2] Di Marzo V. Matias I. (2005). Endocannabinoid control of food intake and energy balance. Nat. Neurosci. 8, 585–589. 10.1038/nn1457 15856067

[B3] Finnerup N. B. Kuner R. Jensen T. S. (2020). Neuropathic pain: from mechanisms to treatment. Physiol. Rev. 101, 259–301. 10.1152/physrev.00045.2019 32584191

[B4] Isensee J. Wenzel C. Buschow R. Weissmann R. Kuss A. W. Hucho T. (2014). Subgroup-elimination transcriptomics identifies signaling proteins that define subclasses of TRPV1-positive neurons and a novel paracrine circuit. PloS One 9, e115731 10.1371/journal.pone.0115731 25551770 PMC4281118

[B17] Jin J. Xie Y. Shi C. Ma J. Wang Y. Qiao L. (2020). Lipoxin A4 inhibits NLRP3 inflammasome activation in rats with non-compressive disc herniation through the JNK1/Beclin-1/PI3KC3 pathway. Front. Neurosci. 14, 799 10.3389/fnins.2020.00799 33071721 PMC7539067

[B5] Laganà P. Anastasi G. Marano F. Piccione S. Singla R. K. Dubey A. K. (2019). Phenolic substances in foods: health effects as anti-inflammatory and antimicrobial agents. J. AOAC Int. 102, 1378–1387. 10.5740/jaoacint.19-0131 31200787

[B6] Li H. Yang T. Tang H. Tang X. Shen Y. Benghezal M. (2019). *Helicobacter pylori* infection is an infectious disease and the empiric therapy paradigm should be changed. Precis. Clin. Med. 2, 77–80. 10.1093/pcmedi/pbz009 PMC8985775 35692450

[B7] Pu Q. Lin P. Wang Z. Gao P. Qin S. Cui L. (2019). Interaction among inflammasome, autophagy and non-coding RNAs: new horizons for drug. Precis. Clin. Med. 2, 166–182. 10.1093/pcmedi/pbz019 31598387 PMC6770284

[B8] Russo E. B. (2016). Clinical endocannabinoid deficiency reconsidered: current research supports the theory in migraine, fibromyalgia, irritable bowel, and other treatment-resistant syndromes. Cannabis Cannabinoid Res. 1, 154–165. 10.1089/can.2016.0009 28861491 PMC5576607

[B9] Santos W. B. R. Melo M. A. O. Alves R. S. De Brito R. G. Rabelo T. K. Prado L. D. S. (2019). p-Cymene attenuates cancer pain via inhibitory pathways and modulation of calcium currents. Phytomedicine 61, 152836 10.1016/j.phymed.2019.152836 31035053

[B10] Singla R. K. Ali M. Kamal M. A. Dubey A. K. (2018). Isolation and characterization of nuciferoic acid, a novel keto fatty acid with hyaluronidase inhibitory activity from cocos nucifera linn. Endocarp. Curr. Top. Med. Chem. 18, 2367–2378. 10.2174/1568026619666181224111319 30582479

[B11] Singla R. K. Agarwal T. Xuefei H. Shen B. (2020). Herbal resources to Combat a progressive and degenerative nervous system disorder—Parkinson's disease. Curr. Drug Targets [Epub ahead of print]. 10.2174/1389450121999201013155202 33050857

[B18] Uddin M. S. Mamun A. A. Rahman M. A. Kabir M. T. Alkahtani S. Alanazi I. S. (2020). Exploring the promise of flavonoids to combat neuropathic pain: from molecular mechanisms to therapeutic implications. Front. Neurosci. 14, 478 10.3389/fnins.2020.00478 32587501 PMC7299068

[B12] Yang P. (2019). Maximizing quality of life remains an ultimate goal in the era of precision medicine: exemplified by lung cancer. Precis. Clin. Med. 2, 8–12. 10.1093/pcmedi/pbz001 PMC8985777 35694702

[B13] Zhang B. Dudley J. Mason C.E. Jacobson P. Pesce S. Lili L. (2020). Improved gastrointestinal health for irritable bowel syndrome with metagenome-guided interventions. Precis. Clin. Med. 3 **,** 136–146. 10.1093/pcmedi/pbaa013 32685241 PMC7327130

